# Homozygote *CRIM1* variant is associated with thiopurine-induced neutropenia in leukemic patients with both wildtype *NUDT15* and *TPMT*

**DOI:** 10.1186/s12967-020-02416-7

**Published:** 2020-07-01

**Authors:** Yoomi Park, Hyery Kim, Heewon Seo, Jung Yoon Choi, Youngeun Ma, Sunmin Yun, Byung-Joo Min, Myung-Eui Seo, Keon Hee Yoo, Hyoung Jin Kang, Ho Joon Im, Ju Han Kim

**Affiliations:** 1 Division of Biomedical Informatics, Seoul National University Biomedical Informatics (SNUBI), Seoul National University College of Medicine, 101 Daehak-ro, Jongno-gu, Seoul, 03080 South Korea; 2 Department of Pediatrics, Asan Medical Center Children’s Hospital, University of Ulsan College of Medicine, 88, Olympic-ro 43-gil, Songpa-gu, Seoul, 05505 South Korea; 3 Princess Margaret Cancer Centre, University Health Network, Toronto, ON M5G 1L7 Canada; 4 Department of Pediatrics, Seoul National University College of Medicine, Seoul, 03080 South Korea; 5 Seoul National University Cancer Research Institute, Seoul, South Korea; 6 Department of Pediatrics, Seoul National University Bundang Hospital, Seoul, South Korea; 7 Department of Pediatrics, Samsung Medical Center, Sungkyunkwan University School of Medicine, Seoul, South Korea; 8 Center for Precision Medicine, Seoul National University Hospital, Seoul, 03080 South Korea

**Keywords:** CRIM1, NUDT15, TPMT, 6-Mercaptopurine, Toxicity, Acute lymphoblastic leukemia

## Abstract

**Background:**

*NUDT15* and *TPMT* variants are strong genetic determinants of thiopurine-induced hematological toxicity that results in therapeutic failure in pediatric acute lymphoblastic leukemia (ALL). However, many patients with both wild-type (WT) *NUDT15* and *TPMT* still suffer from thiopurine toxicity and therapeutic failure.

**Methods:**

Whole-exome sequencing was done for discovery (*N *= 244) and replication (*N *= 76) cohorts. Age- and sex-adjusted multiple regression analyses of both WT patients were performed to identify (*p *< 0.01, *N *= 188 for discovery) and validate (*p *< 0.05, *N *= 52 for replication) candidate variants for the tolerated last-cycle 6-mercaptopurine (6-MP) dose intensity percentage (DIP). Both independent and additive effects of the candidate variants on well-known *NUDT15* and *TPMT* were evaluated by multigene prediction models.

**Results:**

Among the 12 candidate variants from the discovery phase, the rs3821169 variant of the gene encoding Cysteine-Rich Transmembrane BMP Regulator 1 (*CRIM1*) was successfully replicated (*p *< 0.05). It showed high interethnic variability with an impressively high allele frequency in East Asians (*T *= 0.255) compared to Africans (0.001), Americans (0.02), Europeans (0.009), and South Asians (0.05). Homozygote carriers of the *CRIM1* rs3821169 variant (*N *= 12, 5%) showed significantly lower last-cycle 6-MP DIPs in the discovery, replication, and combined cohorts (*p *= 0.025, 0.013, and 0.001, respectively). The traditional two-gene model (*NUDT15* and *TPMT*) for predicting 6-MP DIP < 25% was outperformed by the three-gene model that included *CRIM1*, in terms of the area under the receiver operating characteristic curve (0.734 vs. 0.665), prediction accuracy (0.759 vs. 0.756), sensitivity (0.636 vs. 0.523), positive predictive value (0.315 vs. 0.288), and negative predictive value (0.931 vs. 0.913).

**Conclusions:**

The *CRIM1* rs3821169 variant is suggested to be an independent and/or additive genetic determinant of thiopurine toxicity beyond *NUDT15* and *TPMT* in pediatric ALL.

## Background

The associations of *NUDT15* and *TPMT* genetic variants with 6-mercaptopurine (6-MP) intolerance have been very well established in pediatric acute lymphoblastic leukemia (ALL). In European populations, about 50% of thiopurine-induced severe cytotoxic adverse reactions such as severe neutropenia and leukopenia are explained by *NUDT15* and *TPMT* genetic variants [1]. The Clinical Pharmacogenetics Implementation Consortium (CPIC) [2] publishes practical evidence-based guidelines for the clinical implications of 6-MP based on these two genes, supporting the implementation of pharmacogenetic testing in routine clinical practice [3, 4].

Currently, 6-MP dose is clinically titrated based on the known risk variants of *TPMT* or *NUDT15*. However, a substantial proportion of leukemia patients who have no genetic variation in *NUDT15* or *TPMT* still suffer from life-threatening toxicity, which may result in dose reduction and/or discontinuation of 6-MP and resultant therapeutic failure and relapse. Therefore, further discovery of novel genetic variants other than *NUDT15* and *TPMT* variations is urgently needed for preventing 6-MP toxicity and improving pediatric ALL patient care.

The present study aimed to identify novel genetic variations associated with the 6-MP intolerance in pediatric ALL patients who carry both wild-type (WT) *NUDT15* and *TPMT* by using whole-exome sequencing (WES) technology. We identified and systematically evaluated the deterministic effects of novel candidate variants on a clinically important hematological toxicity indicator: the last-cycle 6-MP dose intensity percentage (DIP) tolerated by pediatric ALL patients.

## Methods

### Subjects

A total of 320 Korean pediatric ALL patients receiving 6-MP treatment during maintenance therapy include discovery (*N *= 244) and replication (*N *= 76) cohorts, recruited from two teaching hospitals [Seoul National University Hospital (SNUH) and Asan Medical Center (AMC)] and three teaching hospitals [SNUH, AMC, and Samsung Medical Center (SMC)] located in Seoul, Korea, respectively (Table 1). The discovery cohort was retrospectively collected and sequenced before February 2018, while the replication cohort was subsequently collected and sequenced from October 2018 to November 2019. All of the selected individuals conformed with the exclusion criteria (i.e., relapse of the disease, stem cell transplantation, Burkitt’s lymphoma, mixed phenotype acute leukemia, infant ALL, or very high risk of ALL). The hematological toxicity was estimated based on the measurement of the tolerated last-cycle 6-MP DIP as the clinical endpoint. The recorded 6-MP dose per square meter of the body surface over a 12-week cycle was used to define the actual administered dose as a percentage of the planned dose as the last-cycle DIP. Since East Asian ancestry requires significantly lower 6-MP dose intensity compared to the other ethnic groups [5], patients who require less than 25% of the protocol planned dose were classified as MP-intolerant groups [6]. We have previously presented a detailed description of the subjects and a summary of the measurements [7]. The present study was approved by the SNUH, AMC, and SMC institutional review boards. Written informed consent was obtained from each participant.

**Table 1:** Clinical characteristics of pediatric acute lymphoblastic leukemia (ALL) subjects who are normal metabolizers (NMs) for both *NUDT15* and *TPMT*

Characteristic	Discovery	Replication	Combined
Number of subjects	188	52	240
Age, years^a^	6.9 ± 4.5	7.4 ± 4.5	7.0 ± 4.5
Sex			
Male	108	29	137
Female	80	23	103
Last-cycle 6-MP dose, mg/m^2^/day			
≤ 10	8.68 ± 1.5 (3)	6.29 ± 2.2 (5)	7.19 ± 2.2 (8)
> 10 and ≤ 15	13.89 ± NA (1)	13.21 ± 1.8 (3)	13.38 ± 1.5 (4)
> 15 and ≤ 25	18.52 ± 3.4 (5)	22.13 ± 1.3 (4)	20.12 ± 3.1 (9)
> 25 and ≤ 35	29.95 ± 3.3 (16)	30.49 ± 0.8 (4)	30.06 ± 3.0 (20)
> 35 and ≤ 45	39.54 ± 3.6 (8)	40.18 ± 2.6 (5)	39.79 ± 3.1 (13)
> 45 and ≤ 60	52.71 ± 4.0 (41)	54.84 ± 2.8 (5)	52.94 ± 3.9 (46)
> 60 and ≤ 80	70.79 ± 6.0 (55)	69.35 ± 5.1 (10)	70.57 ± 5.8 (65)
> 80 and ≤ 100	90.87 ± 5.9 (35)	85.98 ± 5.0 (8)	89.96 ± 6.0 (43)*
> 100	112.67 ± 16.6 (24)	122.66 ± 23.3 (8)	115.17 ± 18.7 (32)
Total	68.44 ± 27.6 (188)	59.99 ± 38.2 (52)	66.61 ± 30.3 (240)

Data are *n*, mean ± SD, or mean ± SD (*N*) values

*6-MP* 6-mercaptopurine, *NA* not available

*p* values are for *t*-tests or ^2^ tests as appropriate. *p < 0.05

^a^Data for age were not available for one subject

### Whole-exome sequencing and primary data analysis

**Fig. 1:**
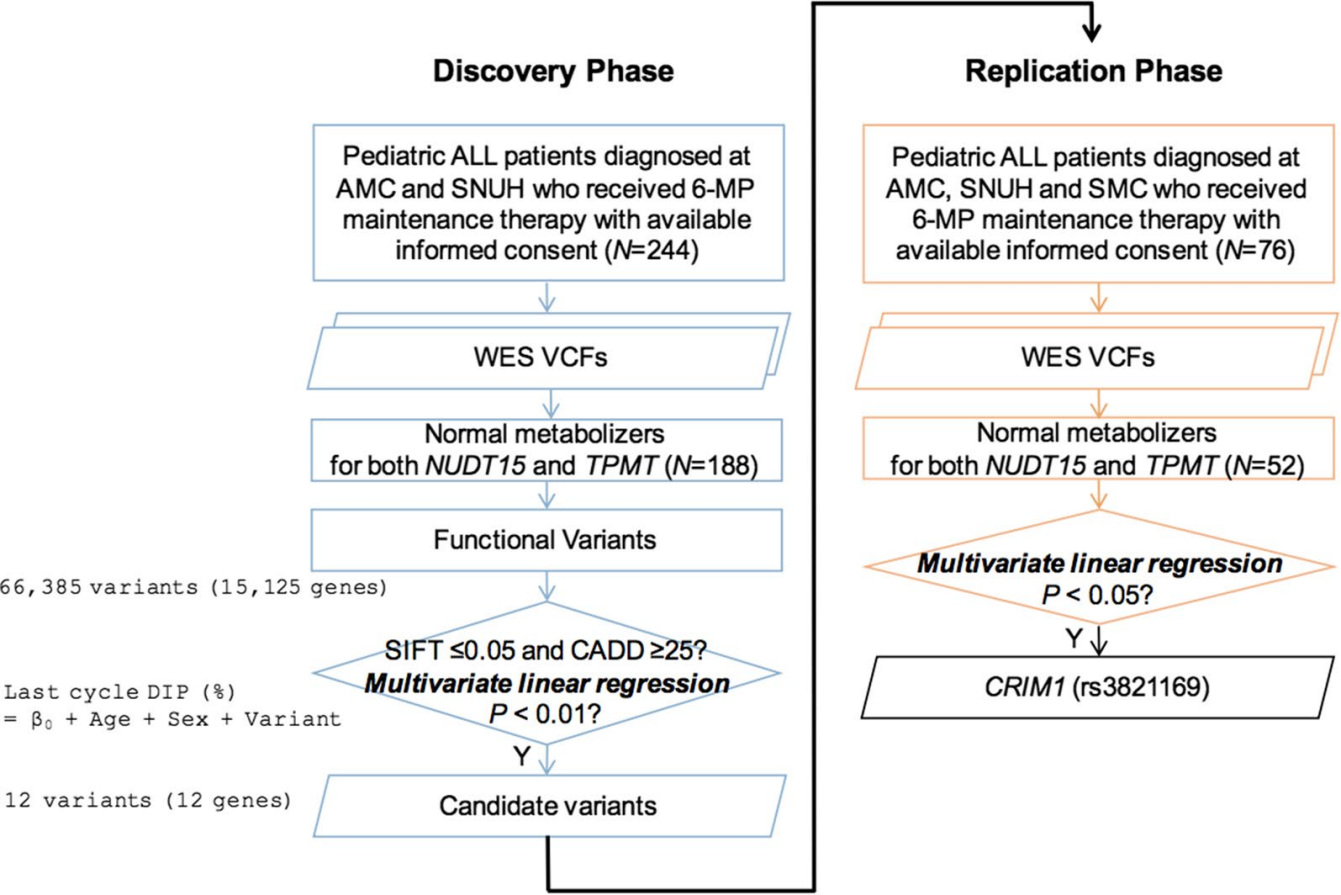
Schematic diagram of the discovery- and replication-phase data analysis steps. *ALL* acute lymphoblastic leukemia, *WES* whole-exome sequencing, *SIFT* sorting intolerant from tolerant, *CADD* combined annotation-dependent depletion, *VCF* variant call format, *DIP* dose intensity percentage, *NM* normal metabolizer, *AMC* Asan Medical Center, *SNUH* Seoul National University Hospital, *SMC* Samsung Medical Center, *6-MP* 6-mercaptopurine

WES data obtained from the 320 pediatric ALL patients were analyzed in a bioinformatics pipeline as we have described previously [7]. Two missense *NUDT15* variants with no officially designated star alleles were confirmed using Sanger sequencing, and false positive variant calls were removed in the further analysis. According to the CPIC guideline updated in February 2019, where the activity of NUDT15*9 for 6-MP was changed from ‘uncertain’ to ‘no function’ [1], one patient was reclassified as a poor metabolizer of *NUDT15*. The present study analyzed the 240 normal metabolizers (188 and 52 in the discovery and replication cohorts, respectively) of both *NUDT15* and *TPMT* according to their star-allele genotypes. In the discovery phase, functional consequences of variants were predicted using SnpEFF (http://snpeff.sourceforge.net) [8], and only variants predicted to have a strong effect on gene function (missense, nonsense, splice-site, frameshift, and in-frame insertion and deletion variants) were chosen (Fig. 1).

Age- and sex-adjusted multivariate linear regression analyses of the DIP model identified 185 variants (*p *< 0.01) in 159 genes, of which 12 candidate variants (in 12 genes, Table 2) determined by 2 in silico prediction methods [i.e., SIFT (sorting intolerant from tolerant) [9] score ≤ 0.05 and CADD (combined annotation-dependent depletion) [10] score ≥ 25] were evaluated in the external replication cohort using multiple regression analyses (Fig. 1). We identified 1 final candidate variant, rs3821169, in the gene encoding Cysteine-Rich Transmembrane BMP Regulator 1 (*CRIM1*) that exhibited statistically significant associations for the last-cycle DIP (*p *< 0.05) in both additive and recessive models. Finally, we performed genotyping assays to experimentally validate the identified candidate variant.

**Table 2:** List of 12 candidate variants for thiopurine toxicity in the discovery cohort (*N *= 188)

SNV: risk allele	Gene symbol	SIFT score	CADD score	ExAC EAS AF	No. of variant carriers	Last-cycle 6-MP DIP (%)			Additive		Recessive	
						Carrier	Noncarrier	ANOVA *p*	Effect size	*p*^†^	Effect size	*p*^†^
rs3821169:T	*CRIM1*	0	25.3	0.243	88	64.41 ± 27.7	71.99 ± 27.0	0.015	− 9.07	0.0079	− 21.09	*0.0248*
rs191083003:T	*FSIP2*	0.01	26.7	3.46E−03	3	25.79 ± 21.1	69.13 ± 27.1	0.007	− 46.98	0.0033	NA	NA
rs67877771:G	*IQCG*	0.04	26.2	0.215	59	61.13 ± 25.3	71.79 ± 28.0	0.010	− 10.68	0.0086	− 22.55	0.2531
rs200125400:A	*SLC22A5*	0	32	2.39E−03	2	16.44 ± 0.7	69.00 ± 27.2	0.007	− 52.63	0.0069	NA	NA
rs141145196:A	*TOP1MT*	0.03	27.1	4.76E−03	2	19.46 ± 17.7	68.97 ± 27.2	0.011	− 54.90	0.0061	NA	NA
rs61758536:A	*SPAG8*	0	26	0.052	28	56.14 ± 26.6	70.60 ± 27.2	0.010	− 14.74	0.0087	NA	NA
rs181036640:A	*DPP7*	0	28.7	0.011	4	31.90 ± 28.4	69.24 ± 27.1	0.007	− 37.27	0.0071	NA	NA
rs34337292:C	*OR9Q2*	0	25.9	0.068	48	59.46 ± 28.7	71.52 ± 26.5	0.002	− 11.98	0.0044	− 37.44	*0.0195*
rs200982819:A	*SLC15A3*	0	29.7	0.028	7	42.40 ± 18.8	69.45 ± 27.4	0.005	− 24.41	0.0056	− 58.42	*0.0340*
rs144612495:T	*GOLGA3*	0.02	25.7	4.00E−03	2	18.16 ± 6.0	68.98 ± 27.2	0.009	− 55.00	0.0049	NA	NA
rs12587478:T	*KLHL33*	0	25	0.059	11	44.20 ± 27.5	69.95 ± 26.9	0.005	− 22.01	0.0034	− 17.75	0.5218
rs746000108:T	*INSR*	0.01	25	5.01E−04	2	18.11 ± 15.8	68.98 ± 27.2	0.009	− 50.77	0.0097	NA	NA

*DIP* dose intensity percentage, *SIFT* sorting intolerant from tolerant, *CADD* combined annotation-dependent depletion, *EAS* East Asians, *AF* allele frequency, *ExAC* Exome Aggregation Consortium, *SNV* single-nucleotide variant; *NA* not available

^**†**^*p* values from multivariate linear regression analyses of additive and recessive models

### Subsequent genotyping and validation

To confirm the genotype calls of the final candidate variant, rs3821169, we performed SNPtype (Fluidigm, San Francisco, CA) assays for 118 subjects with 2 control samples whose blood DNA was available after the WES. In the SNPtype assay, genomic DNA flanking the SNP of interest was amplified by PCR with an STA primer set and Qiagen 2× Multiplex PCR Master Mix (Qiagen) in a total reaction volume of 5 μL that contained 40 ng of genomic DNA. PCR was carried out as follows: 1 cycle at 95 °C for 15 min, and then 14 cycles at 95 °C for 15 s and 60 °C for 4 min. After amplification, STA products were diluted 1:100 in DNA suspension buffer, and 2.5 μL of the diluted STA products was added to a sample premix that contained 3 μL of 2× Fast Probe Master Mix, 0.3 μL of SNPtype 20× Sample Loading Reagent, 0.1 μL of SNPtype Reagent, and 0.036 μL of ROX. After the assay premix and sample premix were loaded into the 192.24 Dynamic Array, the SNPtype assay reaction was carried out as follows: 1 cycle at 95 °C for 5 min; 1 cycle at 95 °C for 15 s, 64 °C for 45 s, and 72 °C for 15 s; 1 cycle at 95 °C for 15 s, 63 °C for 45 s, and 72 °C for 15 s; 1 cycle at 95 °C for 15 s, 62 °C for 45 s, and 72 °C for 15 s; 1 cycle at 95 °C for 15 s, 61 °C for 45 s, and 72 °C for 15 s; 34 cycles at 95 °C for 15 s, 60 °C for 45 s and 72 °C for 15 s; and 1 cycle at 25 °C for 10 s. The genotyping test was carried out using Fluidigm SNP Genotyping Analysis software (version 4.0.1, Fluidigm).

For the two missense variants in *NUDT15* with no officially designated star alleles, we performed independent validation using Sanger sequencing. Exon 1 of *NUDT15*, including the rs780144127 and 13: 48611982 A > G, was amplified for Sanger sequencing. PCR assays were performed directly to amplify 20 ng of the genomic DNA samples to collect the target regions using the oligo-primer pairs. Reaction parameters were as follows: 95 °C for 5 min, followed by 35 cycles of 95 °C for 30 s, 58 °C for 30 s, 72 °C for 1 min and 72 °C for 10 min. RBC HiYield Gel/PCR DNA Mini Kit was used to purify the DNA in the PCR products (Taipai county 220, Taiwan). After purification, the PCR samples were directly sequenced using an ABI 3100 semi-automated sequencing analyzer (Applied Biosystems, Lincoln Center Drive Foster City, CA, USA). The DNA sequences were analyzed using FinchTV version 1.4.0 (Geospiza, Inc., Seattle, WA, USA).

### Single- and multigene prediction accuracies for thiopurine toxicity

Gene-wise variant burden (GVB) analysis was performed to evaluate the aggregated impact of both common and rare variants [7, 11, 12]. The GVB of a coding gene for each individual was defined as the geometric mean of the SIFT scores of the coding variants (SIFT score < 0.7) in the coding gene, where GVB^*G*^ denotes the GVB score of gene *G*. The powers of GVB^*NUDT15*^, GVB^*TPMT*^, and GVB^*CRIM1*^ for predicting the last-cycle 6-MP DIP were systematically evaluated by analyzing ROC (receiver operating characteristic) curves across seven DIP cutoffs (i.e., 15%, 25%, 35%, 45%, 60%, 80%, and 100%) in terms of the areas under the ROC curves (AUCs) in the discovery, replication, and combined cohorts before and after controlling for the effects of the other two genes. Multigene effects were systematically evaluated by defining GVB^*A,B*^ as the geometric mean of GVB^*A*^ and GVB^*B*^.

All statistical analyses were performed using the R statistical package (version 3.5.1). To correctly evaluate the recessive model for the *CRIM1* variant in this study, the effect of the heterozygous rs3821169 variant was ignored when computing GVB^*CRIM1*^.

### Star-allele diplotype vs. gene-wise variant burden

The traditional pharmacogenetic star-allele assignment system classifies study subjects into categorical molecular-phenotype groups. However, novel pharmacogenes do not yet have star-allele assignments. Genes do not work alone, and the categorical nature of traditional star-allele-based molecular phenotyping makes it nontrivial to consistently evaluate the multigene pharmacogenetic effects of a drug. The GVB method assigns a corresponding quantitative score for each gene to each individual, enabling the consistent quantization of multigene GVB scores of an individual into a personalized drug GVB score. To evaluate the clinical utility of the GVB method, we systematically compared the traditional star-allele-based molecular phenotyping method with single- and multigene GVB methods for predicting 6-MP intolerance in pediatric ALL patients (Tables 3 and 4).

**Table 3:** Prediction accuracies of *CRIM1* rs3821169 variant for thiopurine toxicity measured by the tolerated last-cycle 6-MP DIP in pediatric ALL subjects with both wild-type *NUDT15* and *TPMT*

Phase	rs3821169 homozygote carriers	DIP			Sensitivity	Specificity	PPV	NPV	Accuracy
		≤ 25%	> 25%	Total					
Discovery	(+)	2	7	9	0.222	0.961	0.222	0.961	0.926
	(−)	7	172	179					
	Total	9	179	188					
Replication	(+)	3	0	3	0.250	1.000	1.000	0.816	0.827
	(−)	9	40	49					
	Total	12	40	52					
Combined	(+)	5	7	12	0.238	0.968	0.417	0.930	0.904
	(−)	16	212	228					
	Total	21	219	240					

*PPV* positive predictive value, *NPV* negative predictive value

**Table 4:** Comparison of star-allele-based diplotyping vs. the gene-wise variant burden (GVB) method for predicting thiopurine toxicity in pediatric ALL subjects

Phase	Method	Molecular phenotype	Last-cycle 6-MP DIP			Sensitivity	Specificity	PPV	NPV	Accuracy
			≤ 25%	> 25%	Total					
Discovery	CPIC *NUDT15* and *TPMT* metabolizer	PM + IM	10	46	56	0.526	0.796	0.179	0.952	0.775
		NM	9	179	188					
	GVB^*NUDT15,TPMT*^	≤ 0.3	10	42	52	0.526	0.813	0.192	0.953	0.791
		> 0.3	9	183	192					
	GVB^*NUDT15,TPMT,CRIM1*^	≤ 0.3	11	32	43	0.579	0.858	0.256	0.960	0.836
		> 0.3	8	193	201					
		Total	19	225	244					
Replication	CPIC *NUDT15* and *TPMT* metabolizer	PM + IM	13	11	24	0.520	0.784	0.542	0.769	0.697
		NM	12	40	52					
	GVB^*NUDT15,TPMT*^	≤ 0.3	13	10	23	0.520	0.804	0.565	0.774	0.711
		> 0.3	12	41	53					
	GVB^*NUDT15,TPMT,CRIM1*^	≤ 0.45	16	10	26	0.640	0.804	0.615	0.820	0.750
		> 0.45	9	41	50					
		Total	25	51	76					
Combined	CPIC *NUDT15* and *TPMT* metabolizer	PM + IM	23	57	80	0.523	0.794	0.288	0.913	0.756
		NM	21	219	240					
	GVB^*NUDT15,TPMT*^	≤ 0.3	23	52	75	0.523	0.811	0.307	0.914	0.772
		> 0.3	21	224	245					
	GVB^*NUDT15,TPMT,CRIM1*^	≤ 0.45	28	60	88	0.636	0.783	0.318	0.931	0.763
		> 0.45	16	216	232					
		Total	44	276	320					

Prediction accuracies for the last-cycle 6-MP DIP of star-allele-based Clinical Pharmacogenetics Implementation Consortium (CPIC) practice guidelines on *NUDT15* and *TPMT* were compared with the quantitative GVB^*NUDT15,TPMT*^ and GVB^*NUDT15,TPMT,CRIM1*^ methods in the discovery, replication, and combined cohorts. GVB cutoffs were determined by maximizing Youden’s index

*IM* intermediate metabolizer, *PM* poor metabolizer

## Results

### Description of patients

It was determined that 240 of the 320 pediatric ALL patients (188 in the 244 discovery cohort and 52 in the 76 replication cohort) did not carry CPIC-reported pathogenic (or pharmacogenetic) variants in either *NUDT15* or *TPMT*. Table 1 presents the clinical characteristics of the 240 subjects who carried both WT *NUDT15* and *TPMT*. Compared to the non-both-WT subjects (*N *= 80), the both-WT subjects (*N *= 240) demonstrated significantly higher tolerated last-cycle DIPs in the discovery cohort [68.44 ± 27.6 vs. 54.14 ± 29.9 (mean ± SD), *p *= 0.002 by t-test], the replication cohort (59.99 ± 38.2 vs. 33.36 ± 28.7, *p *= 0.001 by *t*-test), and the two cohorts combined. These findings confirm the well-established effects of *NUDT15* and *TPMT* pharmacogenetic variants on thiopurine toxicity in pediatric ALL.

However, Table 1 also demonstrates that 4.8% (9 of 188) and 23.1% (12 of 52) of the both-WT subjects in the discovery and replication cohorts, respectively, were classified as a high-risk group for thiopurine toxicity (DIP < 25%), while 63.8% (120 of 188) and 46.2% (24 of 52), respectively, of the both-WT subjects were classified as a moderate-risk group (DIP < 80%). The difference in the frequency of high-risk subjects between the discovery and replication cohorts is probably due to the lack of available replication data. Overall, 68.8% (*N *= 165) of the 240 subjects who carried both WT *NUDT15* and *TPMT* still demonstrated as-yet-unexplained thiopurine response variability.

### Candidate genes for thiopurine toxicity beyond *NUDT15* and *TPMT*

Age- and sex-adjusted variant-level multivariate linear regression analyses were performed for the 66,385 variants predicted to have strong effects on gene function (i.e., 64,238 missense, 1249 nonsense, 552 splice-site, 332 frameshift, and 4 in-frame insertion and deletion variants) for the both-WT subjects (*N *= 188) in the discovery cohort (*N *= 244) (Fig. 1). Twelve candidate variants in 12 genes were selected by applying a significance cutoff of *p *< 0.01 and 2 in silico prediction methods for variant function (SIFT score ≤ 0.05 and CADD score ≥ 25). Due to the small number of study samples and the rarity of the deleterious variants for full correction of multiple hypotheses, a less-stringent *p* cutoff was applied for the discovery-phase candidate variant analysis.

Table 2 lists the 12 candidate variants for thiopurine toxicity. Only the rs3821169 variant in *CRIM1* was successfully replicated for statistically significant associations with lower last-cycle 6-MP DIP by multivariate regression analyses in both additive (*p *= 0.0483) and recessive (*p *= 0.0132) models (Additional file 1: Table S1). Note that a recessive model could not be correctly applied to 10 of the 12 candidate variants due to the small number of replication subjects along with low allele frequencies (Additional file 1: Table S1).

### Evaluation of the association between the *CRIM1* variant and thiopurine toxicity

**Fig. 2:**
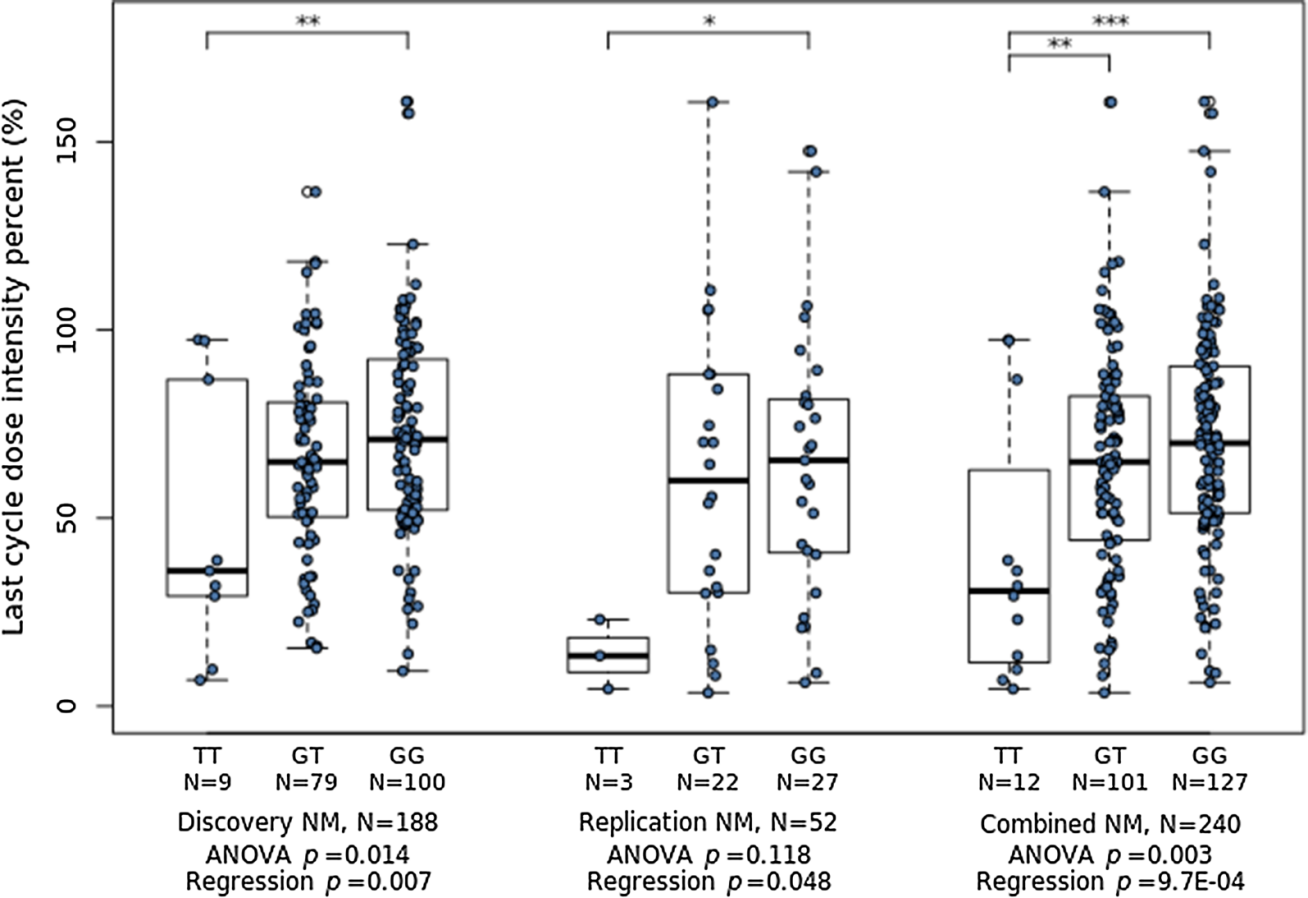
Associations between the *CRIM1* rs3821169 variant and thiopurine toxicity in pediatric ALL subjects with both wild-type (WT) *NUDT15* and *TPMT*. Both ANOVA and multiple linear regression tests identified significant differences in the last-cycle 6-MP DIP among different *CRIM1* rs3821169 genotype groups in the discovery (*p *= 0.014 and 0.007, *N *= 188), replication (p = 0.118 and 0.048, *N *= 52), and combined (p = 0.003 and p = 0.001, *N *= 240) cohorts. *CRIM1*, gene encoding Cysteine-Rich Transmembrane BMP Regulator 1. *p < 0.1, **p < 0.05, ***p < 0.01, post hoc Tukey test

Carriers of the *CRIM1* rs3821169 variant demonstrated significantly lower last-cycle 6-MP DIPs in the discovery cohort (*p *= 0.007), replication cohort (*p *= 0.048), and combined cohort (*p *< 0.001) by multivariate linear regression under an additive model (Fig. 2). Strong associations of this variant under a recessive model were also found for the discovery, replication, and combined cohorts (*p *= 0.025, 0.013, and 0.001, respectively). The statistical power to detect associations in the replication cohort was lost under a dominant model (*p *= 0.028, 0.224, and 0.013), which was at least partly due to the small number of subjects in that cohort. Given the high frequency of *CRIM1* rs3821169 carriers (46.8%) in East Asian subjects, we focused on the homozygote (or recessive) effect of this variant on thiopurine toxicity in the present study.

To evaluate the consistency of the statistical association between the rs3821169 variant and thiopurine toxicity, the candidate variant association was tested across all threshold cutoffs of thiopurine toxicity (i.e., Group 1 (*G*_1_) ≤ 70%, *G*_2_ ≤ 60%, *G*_3_ ≤ 45%, *G*_4_ ≤ 35%, *G*_5_ ≤ 25%, and *G*_6_ < 15% DIPs) by defining two control groups: (1) *G*_0_, comprising the 89, 21, and 110 ALL patients with DIP > 70% in the discovery, replication, and combined cohorts, respectively, and (2) external healthy controls, obtained from the 504 East Asians in the 1000 Genomes Project [13] (Additional file 1: Table S2). Fisher’s exact test for dominant and recessive models and the Cochran–Armitage trend test (CATT) were applied. Four of the six comparison groups for the last-cycle 6-MP DIP in both Fisher’s exact tests (recessive model) and CATTs demonstrated consistent statistical significances for both the *G*_0_ and East-Asian control groups (Additional file 1: Table S2). We experimentally validated and confirmed the rs3821169 genotypes using the Fluidigm genotyping method in 118 subjects for whom blood samples were available, which revealed 97.4% concordance.

### Multigene effects of *NUDT15*, *TPMT*, and *CRIM1* on thiopurine toxicity

**Fig. 3:**
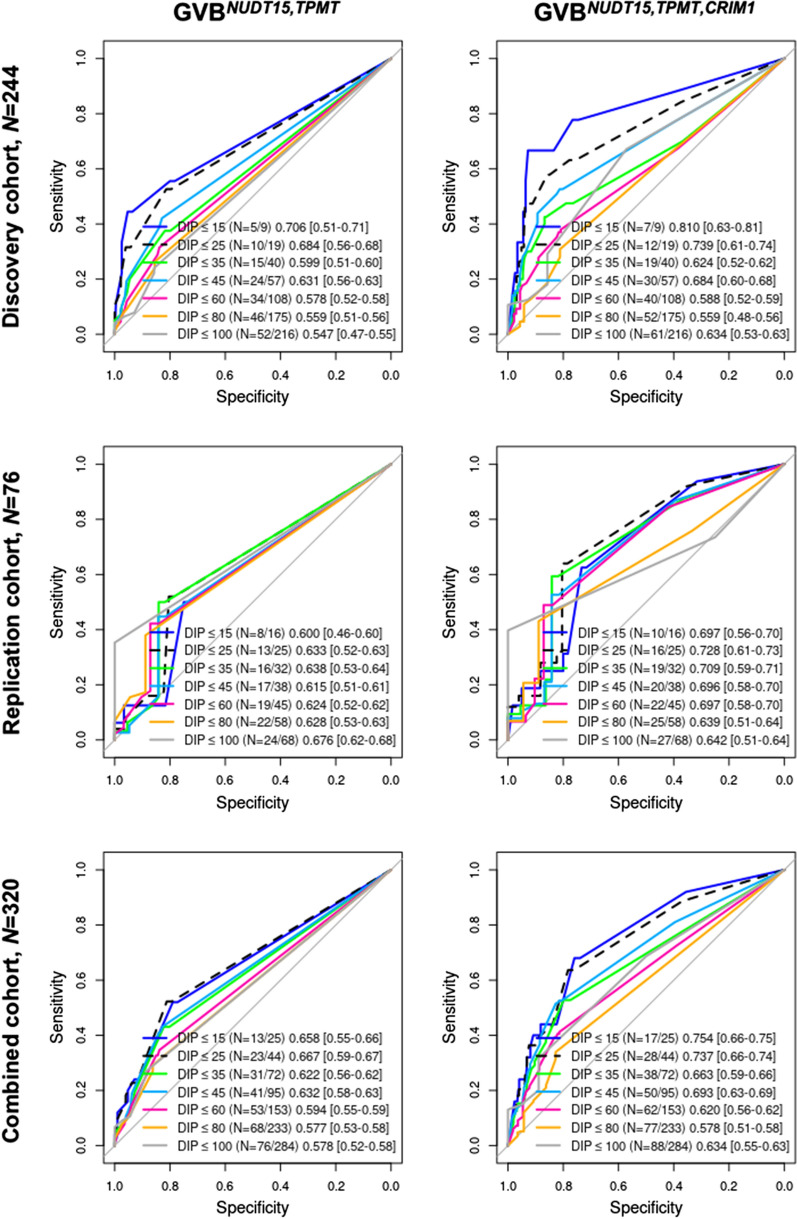
Improvement prediction accuracies for thiopurine toxicity by introducing *CRIM1* into the well-established *NUDT15* and *TPMT* in 320 pediatric ALL subjects. Prediction accuracies (measured in AUCs) for the last-cycle 6-MP DIP of the three-gene model (*NUDT15*, *TPMT* and *CRIM1*) (right panels) outperformed the traditional two-gene model (*NUDT15* and *TPMT*) (left panels) across all seven DIP cutoffs (≤ 15%, ≤ 25%, ≤ 35%, ≤ 45%, ≤ 60%, ≤ 80%, and ≤ 100%) in the discovery (*N *= 244), replication (*N *= 76), and combined (*N *= 320) pediatric ALL cohorts. 95% confidence intervals are in square brackets. GVB, gene-wise variant burden; AUC, area under the receiver operating characteristic curve

To evaluate the additive effects of the novel *CRIM1* rs3821169 variant relative to the well-known *NUDT15* and *TPMT* pharmacogenetic effects, GVB-based ROC analyses were performed before and after introducing the homozygous *CRIM1* rs3821169 variant for the entire cohort of 320 pediatric ALL patients (Fig. 3 and Additional file 1: Figures S1–S3). Figure 3 shows the AUCs representing the diagnostic accuracies of the traditional two-gene prediction model (GVB^*NUDT15,TPMT*^, left panels in the figure) and the newly introduced three-gene prediction model (GVB^*NUDT15,TPMT,CRIM1*^, right panels in the figure) across all seven DIP cutoffs (≤ 15%, ≤ 25%, ≤ 35%, ≤ 45%, ≤ 60%, ≤ 80%, and ≤ 100%) in the discovery (*N *= 244), replication (*N *= 76), and combined (*N *= 320) pediatric ALL cohorts. GVB^*NUDT15,TPMT,CRIM1*^ outperformed the traditional two-gene model GVB^*NUDT15,TPMT*^ at all threshold cutoffs in the discovery, replication, and combined cohorts (e.g., AUC^<15%^ = 0.810 vs. 0.706, 0.697 vs. 0.600, and 0.754 vs. 0.658, respectively; AUC^<25%^ = 0.739 vs. 0.684, 0.728 vs. 0.633, and 0.737 vs. 0.667, respectively), with the only exception being DIP < 100% in the replication cohort (AUC^<100%^ = 0.642 vs. 0.676) (Fig. 3).

More importantly, dose–response relationships for predicting 6-MP intolerance were observed. A lower DIP was associated with a higher AUC for both GVB^*NUDT15,TPMT*^ and GVB^*NUDT15,TPMT,CRIM1*^ (Fig. 3). For example, for the discovery phase of GVB^*NUDT15,TPMT,CRIM1*^, AUC^<15%^ = 0.810 was higher than AUC^<25%^ = 0.739, which was higher than AUC^<35%^ = 0.624 (Fig. 3). Given the high frequency of rs3821169 carriers (46.8%) in East Asian subjects, we focused on the homozygote (or recessive) effect of the *CRIM1* rs3821169 variant on 6-MP intolerance. We defined GVB^*CRIM1*^ as the GVB score of *CRIM1* while ignoring heterozygous rs3821169 and considering only homozygous rs3821169.

### Contributions of single genes to thiopurine toxicity

**Fig. 4:**
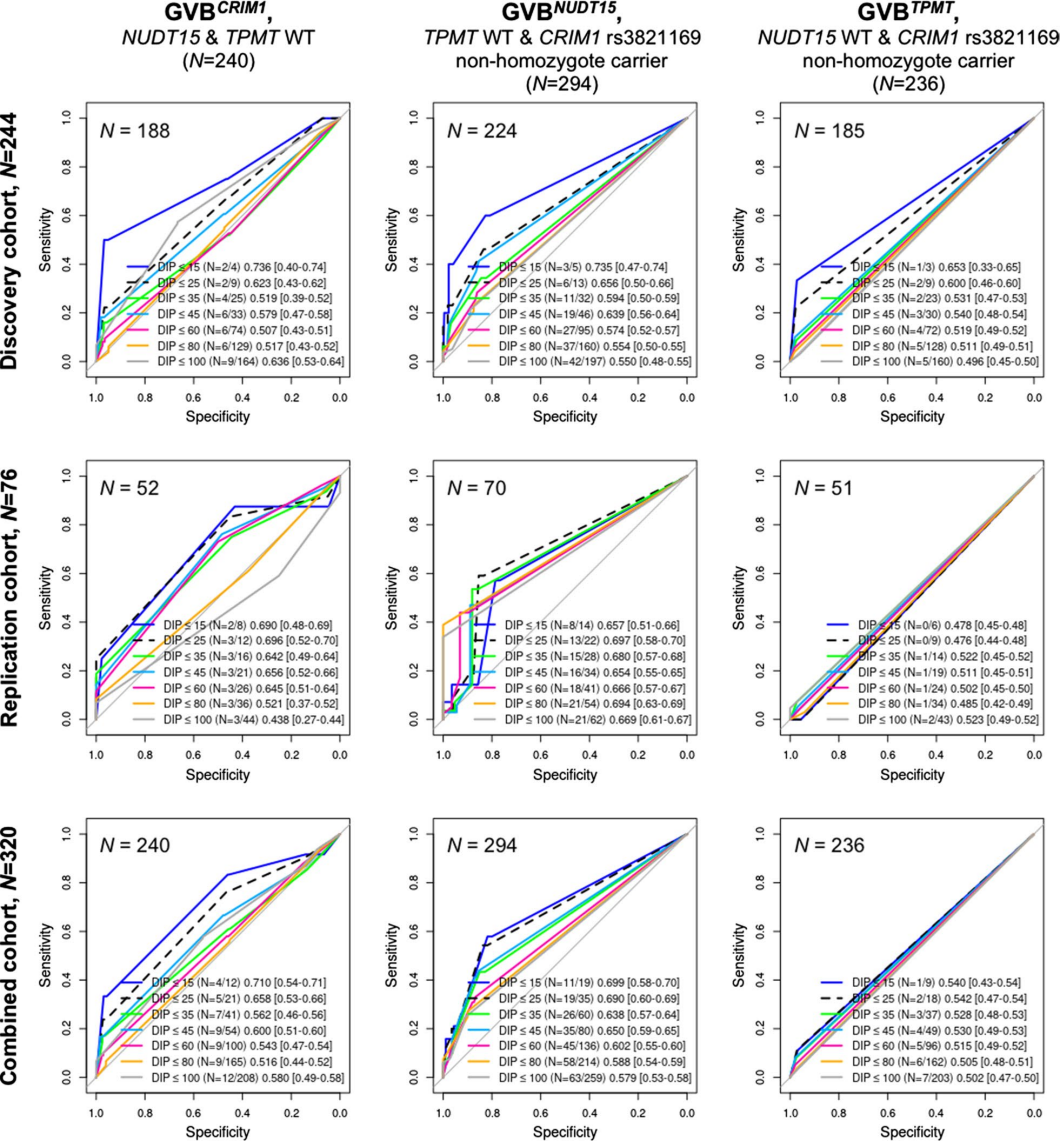
Evaluation of the single-gene contribution of *CRIM1*, *NUDT15*, and *TPMT* in predicting thiopurine toxicity after controlling for the effects of the other two genes in pediatric ALL subjects. Prediction accuracies of GVB^*CRIM1*^, GVB^*NUDT15*^, and GVB^*TPMT*^ for predicting seven cutoffs of the last-cycle 6-MP DIPs (≤ 15%, ≤ 25%, ≤ 35%, ≤ 45%, ≤ 60%, ≤ 80%, and ≤ 100%) were measured using AUCs after controlling for the effects of the other two genes. 95% confidence intervals are in square brackets

Figure 4 demonstrates the diagnostic prediction accuracies for thiopurine toxicity for each of *CRIM1*, *NUDT15*, and *TPMT* after controlling for the effects of the other two genes in the entire cohort (*N *= 320). The AUCs of GVB^*CRIM1*^ were measured for the 240 subjects who carried both WT *NUDT15* and *TPMT* (left panels in Fig. 4), while the AUCs of GVB^*NUDT15*^ were measured for the 294 subjects with WT *TPMT* and nonhomozygote carriers of the *CRIM1* rs3821169 variant (middle panels in Fig. 4). The AUCs of GVB^*TPMT*^ were measured for the 236 subjects with WT *NUDT15* and nonhomozygote carriers of the *CRIM1* rs3821169 variant (right panels in Fig. 4). The prediction accuracies were measured for each of the discovery, replication, and combined cohorts (upper, middle, and lower panels in Fig. 4, respectively).

Overall, *NUDT15* exhibited the best single-gene prediction accuracies for the last-cycle 6-MP DIP for the DIP < 25% cutoff in the discovery (AUC = 0.656, *N *= 224), replication (AUC = 0.697, *N *= 70), and combined (AUC = 0.690, *N *= 294) cohorts. The recessive *CRIM1* model exhibited performances in the discovery (AUC = 0.623, *N *= 188), replication (AUC = 0.696, *N *= 52) and combined (AUC = 0.658, *N *= 240) cohorts that were comparable to *NUDT15*, which is the best-established and strongest predictor of 6-MP intolerance for East Asians. *TPMT* exhibited poor performance in the present study, which can be explained by the very low frequencies of *TPMT* variants in East Asian compared to European populations.

More importantly, each of *NUDT15* and *CRIM1* exhibited a dose–response relationship for predicting thiopurine toxicity. A lower DIP was associated with a higher AUC for both *NUDT15* and *CRIM1* (Fig. 4). Overall, it is suggested that the novel *CRIM1* rs3821169 variant (in its homozygote form) exerts both independent and additive pharmacogenetic effects (to the known *NUDT15* and *TPMT* genes) to thiopurine toxicity, especially in East Asian populations with a high allele frequency (0.243 in the Exome Aggregation Consortium database; Table 2). Additional file 1: Figures S4–S6 provide the results of further detailed analyses of single gene effects on 6-MP intolerance, exhibiting consistent results, as depicted in Fig. 4.

### Evaluation of the prediction accuracies of *NUDT15*, *TPMT*, and *CRIM1*

Table 3 presents the diagnostic accuracies of the *CRIM1* rs3821169 homozygote variant for the last-cycle 6-MP DIP in the discovery (0.926), replication (0.827), and combined (0.904) cohorts. The *CRIM1* rs3821169 homozygosity itself exhibited relatively low sensitivities (0.222–0.250) and positive predictive values (0.222–1.000), and relatively high specificities (0.961–1.000) and negative predictive values (0.816–0.961).

The current CPIC pharmacogenetic testing guideline for 6-MP in treating pediatric ALL patients applies star-allele-based diplotypes of *TPMT* and *NUDT15* [3, 4]. A star allele is defined and/or inferred by a set of genotypes. CPIC guidelines generally do not provide a specific instruction on how to combine multigene interactions for the categorical star-allele classes. Moreover, there are no star-allele assignments for *CRIM1* yet, so evaluating the clinical utility of applying multigene pharmacogenetic testing remains a nontrivial problem. To evaluate the utility of the GVB scoring method for combining multigene effects, we systematically compared the diagnostic accuracies of the traditional star alleles of *NUDT15* and *TPMT* with GVB-quantitation-based GVB^*NUDT15,TPMT*^ as well as GVB^*NUDT15,TPMT,CRIM1*^ (Table 4). The optimal cutoff for the GVB score was determined by maximizing Youden’s index (Additional file 1: Figure S7).

Table 4 demonstrates that GVB^*NUDT15,TPMT*^ yielded slightly better prediction accuracies than the traditional star-allele-based diplotyping method in the discovery (0.791 vs. 0.775), replication (0.711 vs. 0.697), and combined (0.772 vs. 0.756) cohorts, along with improvements in sensitivity, specificity, and positive and negative predictive values. Given that a designated star allele for *CRIM1* is not available yet, we created a three-gene prediction model: GVB^*NUDT15,TPMT,CRIM1*^ outperformed the traditional star-allele-based *NUDT15* and *TPMT* diplotyping method in the discovery (0.836 vs. 0.775), replication (0.750 vs. 0.697), and combined (0.763 vs. 0.756) cohorts, along with exhibiting improvements in sensitivity, specificity, and positive and negative predictive values (Table 4). At the clinical endpoint of the last-cycle 6-MP DIP < 25%, GVB^*NUDT15,TPMT,CRIM1*^ also outperformed the traditional star-allele method in terms of AUC (0.737 vs. 0.665, Fig. 3), prediction accuracy (0.763 vs. 0.756), sensitivity (0.636 vs. 0.523), positive predictive value (0.318 vs. 0.288), and negative predictive value (0.931 vs. 0.913) (Table 4).

GVB^*NUDT15,TPMT,CRIM1*^ also outperformed GVB^*NUDT15,TPMT*^ in the discovery, replication, and combined cohorts in terms of sensitivity (0.579 vs. 0.526, 0.640 vs. 0.520, and 0.636 vs. 0.523, respectively), positive predictive value (0.256 vs. 0.192, 0.615 vs. 0.563, and 0.318 vs. 0.307), and negative predictive value (0.960 vs. 0.953, 0.820 vs. 0.774, 0.931 vs. 0.914). Specificity (0.858 vs. 0.813, 0.804 vs. 0.804, 0.783 vs. 0.811) and accuracy (0.836 vs. 0.791, 0.750 vs. 0.711, and 0.763 vs. 0.772) were improved in the discovery and replication cohorts, but slightly worse in the combined cohort (Table 4). The distribution of nonsynonymous variants in *NUDT15*, *TPMT*, and *CRIM1* genes for 320 ALL patients is summarized in Additional file 1: Table S3.

## Discussion

CRIM1 is a cell-surface transmembrane protein that resembles developmentally important proteins which are known to interact with bone morphogenetic proteins (BMPs). A role of CRIM1 in drug resistance has been suggested by previous studies [14, 15] revealing that the level of mRNA expression of *CRIM1* is high in resistant leukemic cells. This affects the levels of BMPs, suggesting that *CRIM1* regulates the growth and differentiation of hematopoietic cells. The Genomics of Drug Sensitivity in Cancer study [16] found that rs3821169 heterozygous cases showed lower mRNA expression levels compared to the WT cases (Additional file 1: Figure S8, *p *= 0.095 by one-tailed *t*-test). It is suggested that subjects carrying this variant display drug-sensitive responsiveness, although the potential for loss of function of the corresponding protein was not predictable since no homozygous variant was found in the data set, probably due to the low allele frequency of rs3821169 in Western populations. Further experimental validation is needed to determine how *CRIM1* affects thiopurine toxicity.

The present study proposes *CRIM1* as a novel candidate pharmacogenetic gene for predicting thiopurine toxicity in pediatric ALL patients. The last-cycle 6-MP DIP for hematological toxicity measurements was used in estimating the independent and additive pharmacogenetic effects of *CRIM1* over the well-known use of *NUDT15* and *TPMT*. *CRIM1* rs3821169 is a potentially deleterious (SIFT score = 0 and CADD score = 25.3) and very frequent variant in East Asian populations (minor allele frequency = 25%), which increased the predictive power of the present analyses. As expected from the high allele frequency, the homozygous model improved the predictive accuracies for 6-MP intolerance. The heterozygous model demonstrated a moderate phenotypic effect. Recently, a novel association between *CYP2A7* rs73032311 variant and 6-MP-induced leukopenia was reported in subjects with both WT *NUDT15* and *TPMT* [17]. However, in our 240 ALL subjects with both WT *NUDT15* and *TPMT*, the association signal of this variant was not replicated (*p *= 0.891 in age- and sex-adjusted multivariate linear regression analysis of the DIP model). None of the homozygote carriers exhibited DIP < 25% and showed slightly lower DIP (61.51 ± 13.9, *n *= 6) compared to the heterozygote- (68.83 ± 30.3, *n *= 56) and non-carriers (66.08 ± 30.7, *n *= 178). It is suggested that *CYP2A7* rs73032311 may have mild-to-moderate phenotypic effects on 6-MP intolerance only without sufficient clinical utility.

The allele frequency of *CRIM1* rs3821169 (*T *= 0.255) is higher in East Asians than in other racial groups (global = 0.066, Africans = 0.001, Europeans = 0.009, South Asians = 0.05, and Americans = 0.02; Phase 3 of the 1000 Genomes Project [13]). The homozygous carriers of this variant are identified only in the East Asian population (*T *= 0.071). This high interethnic variability might at least partly explain why rs3821169 has not yet been discovered as a biomarker for thiopurine toxicity. The current research bias toward Europeans [18] might have resulted in the statistical power being insufficient for this variant. The inclusion of a large (East Asian) Korean sample treated with 6-MP maintenance therapy (*n *= 320) in the present study allowed us to control the strong and well-known influences of *NUDT15* and *TPMT* by defining the set of both-WT subjects for discovering further biomarkers.

The high interethnic variability of the pharmacogenetic variant is notable. The *NUDT15* rs116855232 variant that was also very recently discovered to be a strong determinant of thiopurine toxicity in a Korean population [19] shows a much higher allele frequency in East Asians (*T *= 0.095) than in other ethnic groups (global = 0.040, Africans = 0.001, Europeans = 0.002, South Asians = 0.07, and Americans = 0.04; Phase 3 of the 1000 Genomes Project [13]). In this study, one rare variant (rs780144127), to which the star allele has not yet been designated, was identified using whole exome sequencing (Additional file 1: Figure S9). The functional effect of this variant on thiopurine toxicity should further be demonstrated, as described in the previous works [20, 21]. Unlike disease-causing genes, pharmacogenes by definition do not exhibit a phenotype unless exposed to the counterpart drug. The lack of overt disadvantageous phenotypes of these pharmacogenes might have permitted high interethnic variability and/or diversity under diverse evolutionary selection pressures in different surroundings.

## Conclusions

In summary, *CRIM1* is a gene associated with 6-MP-induced hematological toxicity. The evidence provided by this study was limited by the insufficient number of samples for the genome-wide significance and the lack of ethnic diversity. Further studies are needed to elucidate the role of *CRIM1* in 6-MP metabolism.

## Supplementary information

**Additional file 1: Table S1.** Evaluation of 12 candidate variants from the discovery cohort (*N* = 188) by using the replication cohort (*N* = 52) for both *NUDT15* and *TPMT* wild-type subjects. **Table S2.** Evaluation of frequency distributions of *CRIM1* rs3821169 genotypes across different cutoffs of the last-cycle 6-mercaptopurine dose intensity percentage tolerated by pediatric acute lymphoblastic leukemia subjects. **Figure S1.** Improvement of prediction accuracy of GVB^*NUDT15,TPMT*^ for thiopurine toxicity after controlling for homozygote carriers of *CRIM1* rs3821169. **Figure S2.** Prediction accuracies of GVB^*NUDT15,CRIM1*^ and GVB^*TPMT,CRIM1*^ for thiopurine toxicity in pediatric ALL subjects. **Figure S3.** Prediction accuracies of GVB^*NUDT15,TPMT,CRIM1*^ for thiopurine toxicity in pediatric ALL subjects. **Figure S4.** Prediction accuracy of GVB^*CRIM1*^ for thiopurine toxicity in pediatric ALL subjects. **Figure S5.** Prediction accuracy of GVB^*NUDT15*^ for thiopurine toxicity in pediatric ALL subjects. **Figure S6.** Prediction accuracy of GVB^*TPMT*^ for thiopurine toxicity in pediatric ALL subjects. **Figure S7.** Youden’s index to find the optimal thresholds for GVB^*NUDT15,TPMT*^ and GVB^*NUDT15,TPMT,CRIM1*^. **Figure S8.** Comparison of *CRIM1* mRNA expression levels of rs3821169 carriers and noncarriers in hematopoietic and lymphoid tissue. **Figure S9.** Results of Sanger sequencing for the two *NUDT15* variants identified via whole exome sequencing.

## Data Availability

All data generated or analyzed during this study are included in this article. If any additional information is required, it may be obtained by request from the corresponding author.

## Abbreviations

6-MP: 6-Mercaptopurine
ALL: Acute lymphoblastic leukemia
WES: Whole-exome sequencing
CPIC: Clinical Pharmacogenetics Implementation Consortium
WT: Wild type
DIP: Dose intensity percentage
SNUH: Seoul National University Hospital
AMC: Asan Medical Center
SMC: Samsung Medical Center
NM: Normal metabolizer
GVB: Gene-wise variant burden
SIFT: Sorting intolerant from tolerant
CADD: Combined annotation-dependent depletion
ROC: Receiver operating characteristic
AUC: Area under the ROC curve
CATT: Cochran–Armitage trend test
CRIM1: Cysteine Rich Transmembrane BMP Regulator 1
BMP: Bone morphogenetic protein
