# Supplementary material for: Homozygote CRIM1 variant is associated with thiopurine-induced neutropenia in leukemic patients with both wildtype NUDT15 and TPMT
Source: J Transl Med. 2020 Jul 1;18:265. doi: 10.1186/s12967-020-02416-7 (PMC7328279; doi:10.1186/s12967-020-02416-7)
Supplement: Supplementary file 1 — Additional file 1: Table S1. Evaluation of 12 candidate variants from the discovery cohort (N = 188) by using the replication cohort (N = 52) for both NUDT15 and TPMT wild-type subjects. Table S2. Evaluation of frequency distributions of CRIM1 rs3821169 genotypes across different cutoffs of the last-cycle 6-mercaptopurine dose intensity percentage tolerated by pediatric acute lymphoblastic leukemia subjects. Figure S1. Improvement of prediction accuracy of GVBNUDT15,TPMT for thiopurine toxicity after controlling for homozygote carriers of CRIM1 rs3821169. Figure S2. Prediction accuracies of GVBNUDT15,CRIM1 and GVBTPMT,CRIM1 for thiopurine toxicity in pediatric ALL subjects. Figure S3. Prediction accuracies of GVBNUDT15,TPMT,CRIM1 for thiopurine toxicity in pediatric ALL subjects. Figure S4. Prediction accuracy of GVBCRIM1 for thiopurine toxicity in pediatric ALL subjects. Figure S5. Prediction accuracy of GVBNUDT15 for thiopurine toxicity in pediatric ALL subjects. Figure S6. Prediction accuracy of GVBTPMT for thiopurine toxicity in pediatric ALL subjects. Figure S7. Youden’s index to find the optimal thresholds for GVBNUDT15,TPMT and GVBNUDT15,TPMT,CRIM1. Figure S8. Comparison of CRIM1 mRNA expression levels of rs3821169 carriers and noncarriers in hematopoietic and lymphoid tissue. Figure S9. Results of Sanger sequencing for the two NUDT15 variants identified via whole exome sequencing. [file 12967_2020_2416_MOESM1_ESM.docx]

**Table S1. Evaluation of 12 candidate variants from the discovery cohort (*N*=188) by using the replication cohort (*N*=52) for both *NUDT15* and *TPMT* wild-type subjects.**

| **SNV:**  **risk allele** | **Gene symbol** | **SIFT score** | **CADD score** | **ExAC EAS AF** | **No. of**  **variant carriers** | **Last cycle 6-MP DIP (%)** | | | **Additive** | | **Recessive** | |
| --- | --- | --- | --- | --- | --- | --- | --- | --- | --- | --- | --- | --- |
|  |  |  |  |  |  | **Carrier** | **Noncarrier** | **ANOVA *p*** | **Effect Size** | ***p^†^*** | **Effect size** | ***p^†^*** |
| rs3821169:T | *CRIM1* | 0 | 25.3 | 0.243 | 25 | 55.14±40.5 | 64.47±36.1 | 0.118 | –16.55 | **0.0483** | –52.27 | **0.0132** |
| rs191083003:T | *FSIP2* | 0.01 | 26.7 | 3.46E-03 | 1 | 8.82±NA | 60.99±37.8 | 0.178 | –39.52 | 0.2877 | NA | NA |
| rs67877771:G | *IQCG* | 0.04 | 26.2 | 0.215 | 17 | 65.98±24.5 | 57.07±43.3 | 0.676 | 0.52 | 0.9492 | –16.54 | 0.3899 |
| rs200125400:A | *SLC22A5* | 0 | 32 | 2.39E-03 | 2 | 56.21±37.1 | 60.14±38.6 | 0.888 | 14.29 | 0.5975 | NA | NA |
| rs141145196:A | *TOP1MT* | 0.03 | 27.1 | 4.76E-03 | 1 | 142.05±NA | 58.38±36.7 | **0.028** | 68.60 | 0.0626 | NA | NA |
| rs61758536:A | *SPAG8* | 0 | 26 | 0.052 | 5 | 42.83±37.6 | 61.81±38.2 | 0.295 | –17.38 | 0.3186 | NA | NA |
| rs181036640:A | *DPP7* | 0 | 28.7 | 0.011 | 1 | 88.24±NA | 59.43±38.3 | 0.460 | 41.32 | 0.2688 | NA | NA |
| rs34337292:C | *OR9Q2* | 0 | 25.9 | 0.068 | 14 | 53.67±28.9 | 62.31±41.2 | 0.474 | –5.15 | 0.6550 | NA | NA |
| rs200982819:A | *SLC15A3* | 0 | 29.7 | 0.028 | 1 | 80.13±NA | 59.59±38.4 | 0.599 | 11.78 | 0.7521 | NA | NA |
| rs144612495:T | *GOLGA3* | 0.02 | 25.7 | 4.00E-03 | 1 | 88.24±NA | 59.43±38.3 | 0.460 | 41.32 | 0.2688 | NA | NA |
| rs12587478:T | *KLHL33* | 0 | 25 | 0.059 | 2 | 30.82±1.2 | 61.15±38.5 | 0.275 | –20.96 | 0.4436 | NA | NA |
| rs746000108:T | *INSR* | 0.01 | 25 | 5.01E-04 | 0 | NA | NA | NA | NA | NA | NA | NA |

**^†^***p* values from multivariate linear regression analyses of additive and recessive models; DIP, dose intensity percentage; SIFT, sorting intolerant from tolerant; CADD, combined annotation-dependent depletion; EAS, East Asians; AF, allele frequency; ExAC, Exome Aggregation Consortium; SNV, single-nucleotide variant; NA, Not Available.

**Table S2. Evaluation of frequency distributions of *CRIM1* rs3821169 genotypes across different cutoffs of the last-cycle 6-mercaptopurine dose intensity percentage tolerated by pediatric acute lymphoblastic leukemia subjects.**

| Type | Group | Discovery phase (WES) | | | | | | | | Group | Comparison with the 1000 Genomes EAS (*N*=504) | | | | | | | |
| --- | --- | --- | --- | --- | --- | --- | --- | --- | --- | --- | --- | --- | --- | --- | --- | --- | --- | --- |
|  |  | REF | HET | HOM | Dominant | | Recessive | | CATT |  | REF | HET | HOM | Dominant | | Recessive | | CATT |
|  |  |  |  |  | *p* | OR  (95% CI) | *p* | OR  (95% CI) | *p* |  |  |  |  | *p* | OR  (95% CI) | *p* | OR  (95% CI) | *p* |
| Combined | *G_0>_*70 | 62 | 45 | 3 |  |  |  |  |  | EAS | 283 | 185 | 36 |  |  |  |  |  |
|  | *G_1_*≤70 | 65 | 56 | 9 | 0.364 | 1.29 (0.8-2.2) | 0.234 | 2.64 (0.6-15.6) | 0.168 | *G_1_*≤70 | 65 | 56 | 9 | 0.236 | 1.28 (0.9-1.9) | 1.000 | 0.97 (0.4-2.1) | 0.335 |
|  | *G_2_*≤60 | 49 | 42 | 9 | 0.333 | 1.34 (0.8-2.4) | 0.073 | 3.51 (0.8-20.7) | 0.102 | *G_2_*≤60 | 49 | 42 | 9 | 0.226 | 1.33 (0.8-2.1) | 0.532 | 1.29 (0.5-2.8) | 0.192 |
|  | *G_3_*≤45 | 19 | 26 | 9 | **0.013** | 2.37 (1.2-5.0) | **0.002** | 7.04 (1.7-42.3) | **7.15E-04** | *G_3_*≤45 | 19 | 26 | 9 | **0.004** | 2.36 (1.3-4.5) | **0.030** | 2.59 (1-5.9) | **8.79E-04** |
|  | *G_4_*≤35 | 14 | 20 | 7 | **0.018** | 2.48 (1.1-5.7) | **0.004** | 7.22 (1.5-45.6) | **0.001** | *G_4_*≤35 | 14 | 20 | 7 | **0.009** | 2.47 (1.2-5.2) | **0.034** | 2.67 (0.9-6.7) | **0.002** |
|  | *G_5_*≤25 | 8 | 8 | 5 | 0.154 | 2.09 (0.7-6.3) | **0.003** | 10.8 (1.9-76.4) | **0.007** | *G_5_*≤25 | 8 | 8 | 5 | 0.119 | 2.08 (0.8-5.9) | **0.018** | 4.04 (1.1-12.4) | **0.014** |
|  | *G_6_*≤15 | 4 | 4 | 4 | 0.142 | 2.56 (0.6-12.3) | **0.002** | 16.88 (2.4-136) | **0.003** | *G_6_*≤15 | 4 | 4 | 4 | 0.145 | 2.56 (0.7-11.8) | **0.010** | 6.45 (1.4-25.5) | **0.008** |
| Discovery phase | *G_0>_*70 | 51 | 35 | 3 |  |  |  |  |  | EAS | 283 | 185 | 36 |  |  |  |  |  |
|  | *G_1_*≤70 | 49 | 44 | 6 | 0.308 | 1.37 (0.7-2.5) | 0.503 | 1.84 (0.4-11.7) | 0.221 | *G_1_*≤70 | 49 | 44 | 6 | 0.227 | 1.31 (0.8-2.1) | 0.831 | 0.84 (0.3-2.1) | 0.416 |
|  | *G_2_*≤60 | 37 | 31 | 6 | 0.430 | 1.34 (0.7-2.6) | 0.302 | 2.52 (0.5-16.1) | 0.202 | *G_2_*≤60 | 37 | 31 | 6 | 0.381 | 1.28 (0.8-2.2) | 0.810 | 1.15 (0.4-2.9) | 0.363 |
|  | *G_3_*≤45 | 10 | 17 | 6 | **0.014** | 3.06 (1.2-8.1) | **0.012** | 6.25 (1.2-41.3) | **0.001** | *G_3_*≤45 | 10 | 17 | 6 | **0.006** | 2.94 (1.3-7.1) | **0.036** | 2.88 (0.9-7.8) | **0.001** |
|  | *G_4_*≤35 | 8 | 13 | 4 | **0.040** | 2.83 (1-8.4) | **0.040** | 5.35 (0.8-39.4) | **0.006** | *G_4_*≤35 | 8 | 13 | 4 | **0.023** | 2.72 (1.1-7.4) | 0.111 | 2.47 (0.6-7.9) | **0.011** |
|  | *G_5_*≤25 | 3 | 4 | 2 | 0.292 | 2.66 (0.5-17.5) | 0.065 | 7.85 (0.6-81.6) | **0.039** | *G_5_*≤25 | 3 | 4 | 2 | 0.193 | 2.56 (0.5-16) | 0.138 | 3.7 (0.4-20.4) | 0.074 |
|  | *G_6_*≤15 | 2 | 0 | 2 | 1 | 1.34 (0.1-19.2) | **0.013** | 25.41 (1.4-472.8) | **0.049** | *G_6_*≤15 | 2 | 0 | 2 | 1 | 1.28 (0.1-17.8) | **0.030** | 12.84 (0.9-181.8) | 0.072 |
| Replication phase | *G_0>_*70 | 11 | 10 | 0 |  |  |  |  |  | EAS | 283 | 185 | 36 |  |  |  |  |  |
|  | *G_1_*≤70 | 16 | 12 | 3 | 1 | 1.03 (0.3-3.6) | 0.264 | Inf (0.3-Inf) | 0.604 | *G_1_*≤70 | 16 | 12 | 3 | 0.710 | 1.2 (0.5-2.7) | 0.486 | 1.39 (0.3-4.9) | 0.543 |
|  | *G_2_*≤60 | 12 | 11 | 3 | 0.772 | 1.28 (0.3-4.7) | 0.242 | Inf (0.3-Inf) | 0.369 | *G_2_*≤60 | 12 | 11 | 3 | 0.321 | 1.49 (0.6-3.6) | 0.428 | 1.69 (0.3-6) | 0.256 |
|  | *G_3_*≤45 | 9 | 9 | 3 | 0.758 | 1.45 (0.4-5.9) | 0.232 | Inf (0.4-Inf) | 0.246 | *G_3_*≤45 | 9 | 9 | 3 | 0.266 | 1.71 (0.6-4.7) | 0.199 | 2.16 (0.4-7.9) | 0.146 |
|  | *G_4_*≤35 | 6 | 7 | 3 | 0.508 | 1.80 (0.4-8.5) | 0.072 | Inf (0.6-Inf) | 0.128 | *G_4_*≤35 | 6 | 7 | 3 | 0.200 | 2.13 (0.7-7.2) | 0.111 | 2.99 (0.5-11.6) | 0.059 |
|  | *G_5_*≤25 | 5 | 4 | 3 | 0.721 | 1.52 (0.3-8.3) | **0.040** | Inf (0.8-Inf) | 0.141 | *G_5_*≤25 | 5 | 4 | 3 | 0.384 | 1.79 (0.5-7.3) | 0.055 | 4.31 (0.7-18.3) | 0.080 |
|  | *G_6_*≤15 | 2 | 4 | 2 | 0.238 | 3.17 (0.4-39.2) | 0.069 | Inf (0.5-Inf) | **0.049** | *G_6_*≤15 | 2 | 4 | 2 | 0.147 | 3.83 (0.7-39.2) | 0.113 | 4.31 (0.4-25.3) | **0.029** |

**Table S3. The raw genotype of nonsynonymous variants in *NUDT15*, *TPMT*, and *CRIM1* for 320 ALL patients.**

| **Gene** |  | ***CRIM1*** | | | | ***NUDT15*** | | | | | | ***TPMT*** | | **Total (DIP < 25), *N*=320** |
| --- | --- | --- | --- | --- | --- | --- | --- | --- | --- | --- | --- | --- | --- | --- |
| **rsID** |  | rs3821169 | rs757223335 | rs951475238 | rs572679851 | rs780144127 | rs746071566 | rs869320766 | rs186364861 | rs116855232 | rs147390019 | rs1142345 | rs75543815 |  |
| **Associated star allele** |  | . | . | . | . | . | *9 | *2,*6 | *5 | *2,*3 | *4 | *3A,*3C | *6 |  |
| **SIFT** |  | 0 | 0.07 | 0.16 | 0.18 | 0.12 | . | . | 0.02 | 0.09 | 0.12 | 0.01 | 0.78 |  |
|  | Poor or Intermediate  Metabolizers | + |  |  |  |  |  | + |  | + |  |  |  | 2 (1) |
|  |  | + |  |  |  |  |  |  |  | + |  |  |  | 16 (6) |
|  |  |  | + |  |  |  |  |  |  | + |  |  |  | 1 (1) |
|  |  | ++ |  |  |  |  |  |  |  | + |  |  |  | 1 (0) |
|  |  |  |  |  |  |  |  | ++ |  | ++ |  |  |  | 1 (1) |
|  |  | + |  |  |  |  |  |  |  | ++ |  |  |  | 1 (1) |
|  |  | + |  |  |  |  |  | + |  |  |  |  |  | 1 (0) |
|  |  |  |  |  |  |  |  |  |  | + |  |  |  | 24 (6) |
|  |  |  |  |  |  |  |  |  |  |  | + |  |  | 2 (0) |
|  |  | + |  |  |  |  |  |  |  |  | + |  |  | 2 (0) |
|  |  |  |  |  |  |  |  | + |  |  |  |  |  | 2 (1) |
|  |  | + |  |  |  |  |  | + |  | ++ |  |  |  | 1 (1) |
|  |  |  |  |  |  |  |  | + |  | + |  |  |  | 7 (1) |
|  |  |  |  |  |  |  |  |  | + |  |  |  |  | 3 (0) |
|  |  |  |  |  |  | + |  |  |  | + |  |  |  | 1 (0) |
|  |  | + |  |  |  |  |  |  | + |  |  |  |  | 2 (0) |
|  |  | ++ |  |  |  |  |  | + |  | + |  |  |  | 3 (1) |
|  |  |  |  |  |  |  | + |  |  |  |  |  |  | 1 (1) |
|  |  |  |  |  |  |  |  |  |  | + |  |  | + | 1 (0) |
|  |  |  |  |  |  |  |  |  |  |  |  | + |  | 3 (0) |
|  |  | + |  |  |  |  |  |  |  |  |  | + |  | 5 (2) |
|  | Normal Metabolizers | ++ |  |  |  |  |  |  |  |  |  |  |  | 12 (5) |
|  |  | + |  |  |  |  |  |  |  |  |  |  |  | 100 (8) |
|  |  | + |  |  | + |  |  |  |  |  |  |  |  | 1 (0) |
|  |  |  |  | + |  |  |  |  |  |  |  |  |  | 1 (0) |
|  |  |  |  |  |  |  |  |  |  |  |  |  |  | 126 (8) |
|  |  |  |  |  |  |  |  |  |  |  |  |  | **Total** | 320 (44) |

+: heterozygous variant; ++: homozygous variant

**Figure S1. Improvement of prediction accuracy of GVB*^NUDT15,TPMT^* for thiopurine toxicity after controlling for homozygote carriers of *CRIM1* rs3821169.** Diagnostic accuracies were measured by AUCs before (left panel, *N*=320) and after (right panel, *N*=303) controlling homozygote rs3821169 carriers across different cutoffs of the tolerated last-cycle 6-mercaptopurine dose intensity percentage (%) in discovery, replication, and combined pediatric acute lymphoblastic leukemia cohorts.


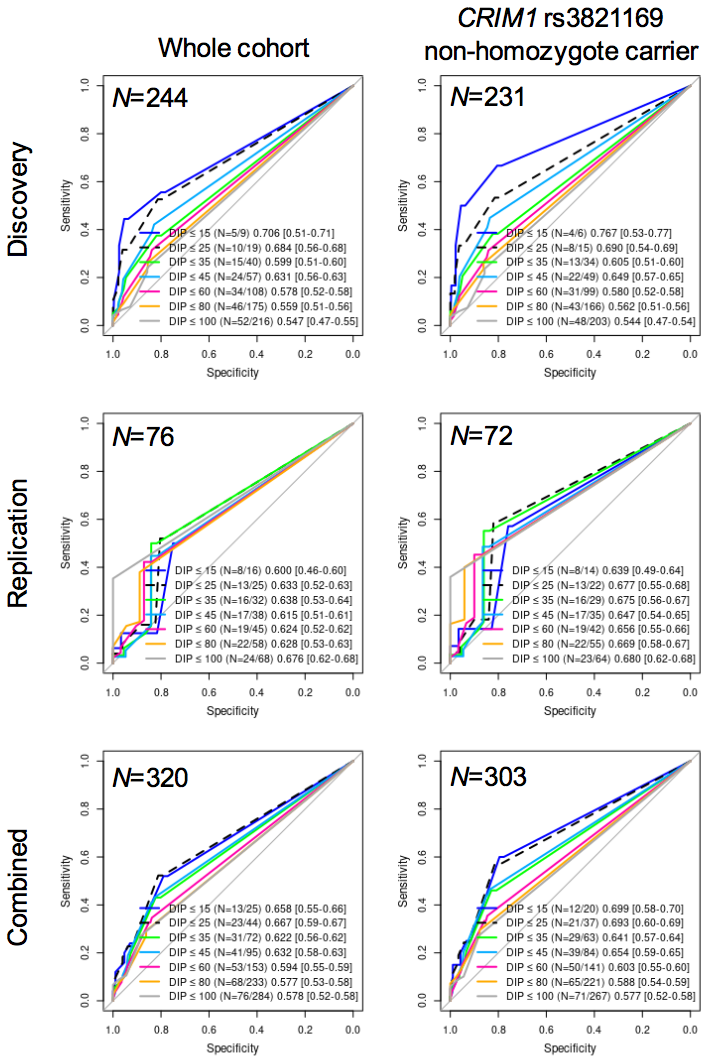


**Figure S2. Prediction accuracies of GVB*^NUDT15,CRIM1^* and GVB*^TPMT,CRIM1^* for thiopurine toxicity in pediatric ALL subjects.** Diagnostic accuracies of two-gene models were measured by AUCs before and after controlling for the other gene across different cutoffs of the tolerated last-cycle 6-mercaptopurine dose intensity percentage (%) in discovery, replication, and combined pediatric ALL cohorts.


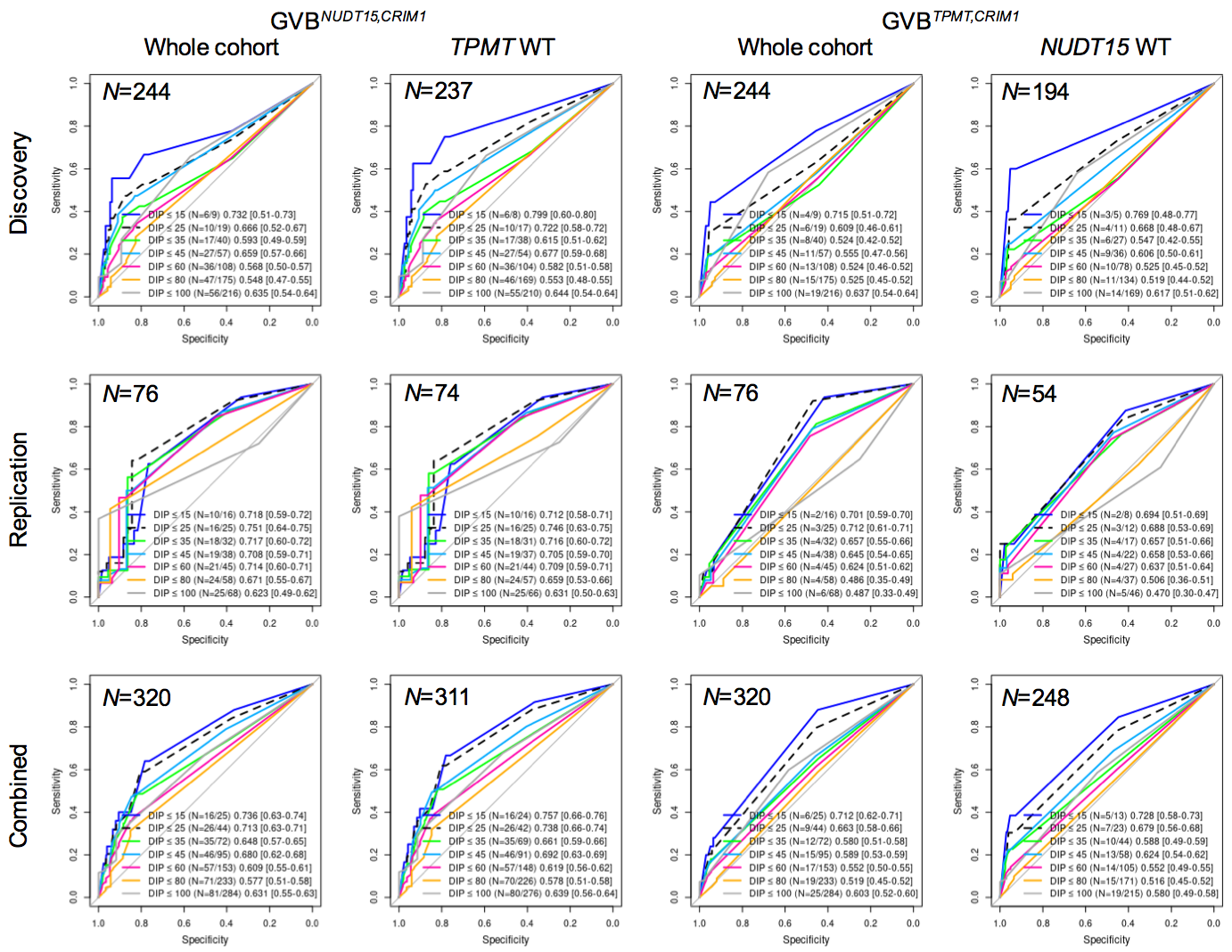


**Figure S3. Prediction accuracies of GVB*^NUDT15,TPMT,CRIM1^* for thiopurine toxicity in pediatric ALL subjects.** Diagnostic accuracies of GVB*^NUDT15,TPMT,CRIM1^* were measured by AUCs with or without controlling for the effect of other confounding variants across different cutoffs of the tolerated last-cycle 6-mercaptopurine dose intensity percentage (%) in discovery, replication, and combined cohorts.


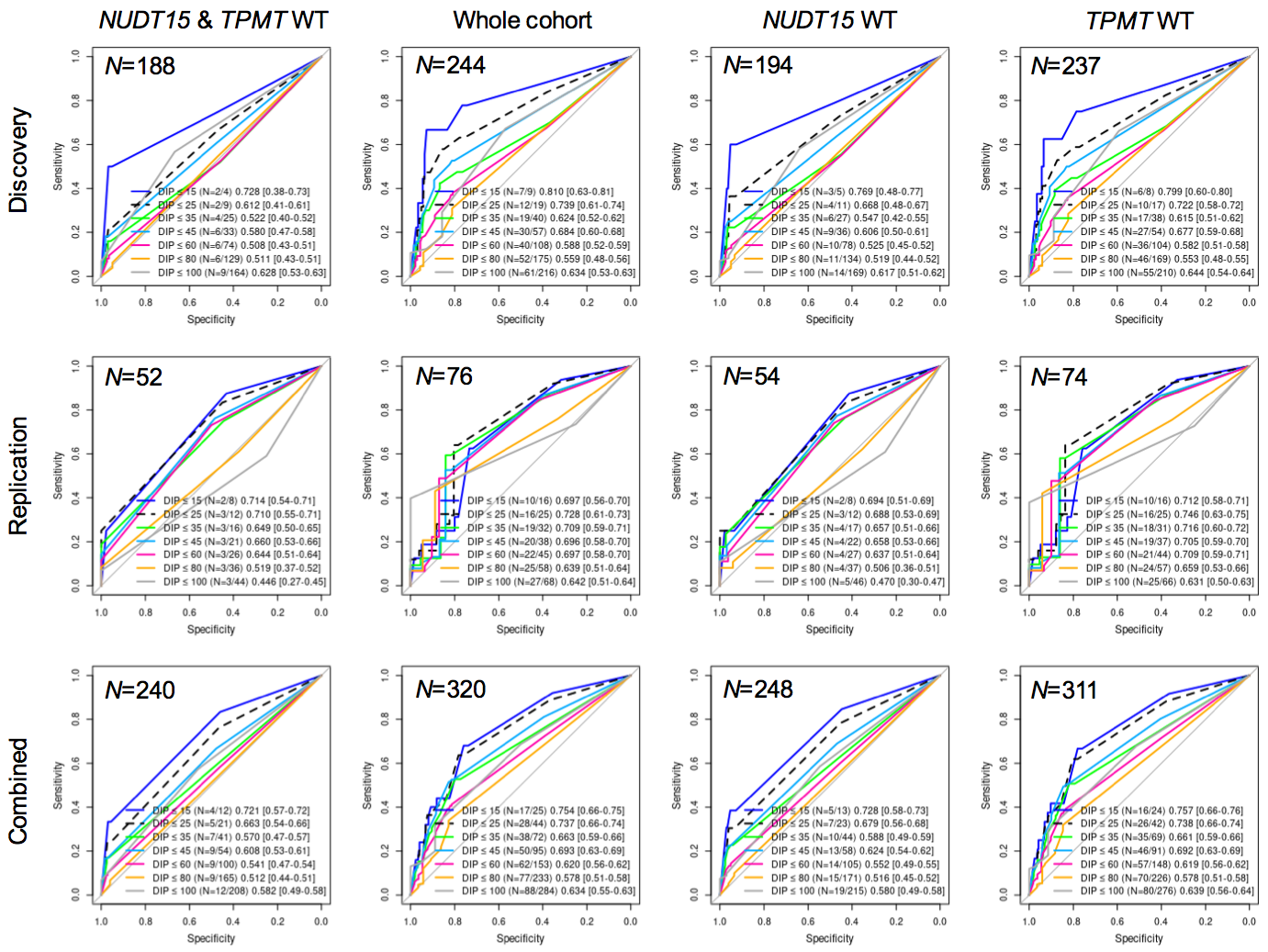


**Figure S4. Prediction accuracy of GVB*^CRIM1^* for thiopurine toxicity in pediatric ALL subjects.** Diagnostic accuracies were measured by AUCs with or without controlling for the effect of other confounding variants across different cutoffs of the tolerated last-cycle 6-mercaptopurine dose intensity percentage (%) in discovery, replication, and combined cohorts.


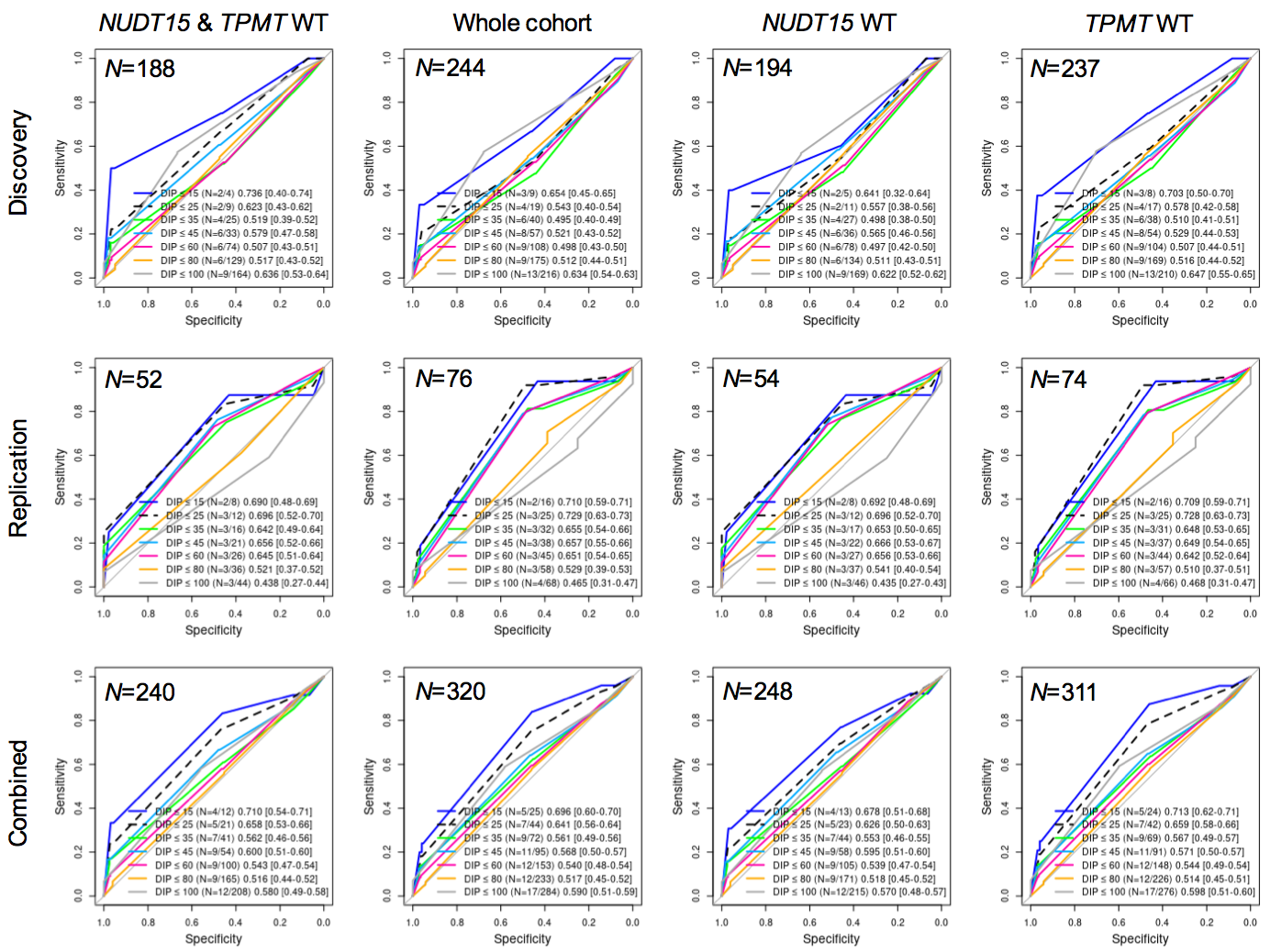


**Figure S5. Prediction accuracy of GVB*^NUDT15^* for thiopurine toxicity in pediatric ALL subjects.** Diagnostic accuracies were measured by AUCs with or without controlling for the effect of other confounding variants across different cutoffs of the tolerated last-cycle 6-mercaptopurine dose intensity percentage (%) in discovery, replication, and combined cohorts.


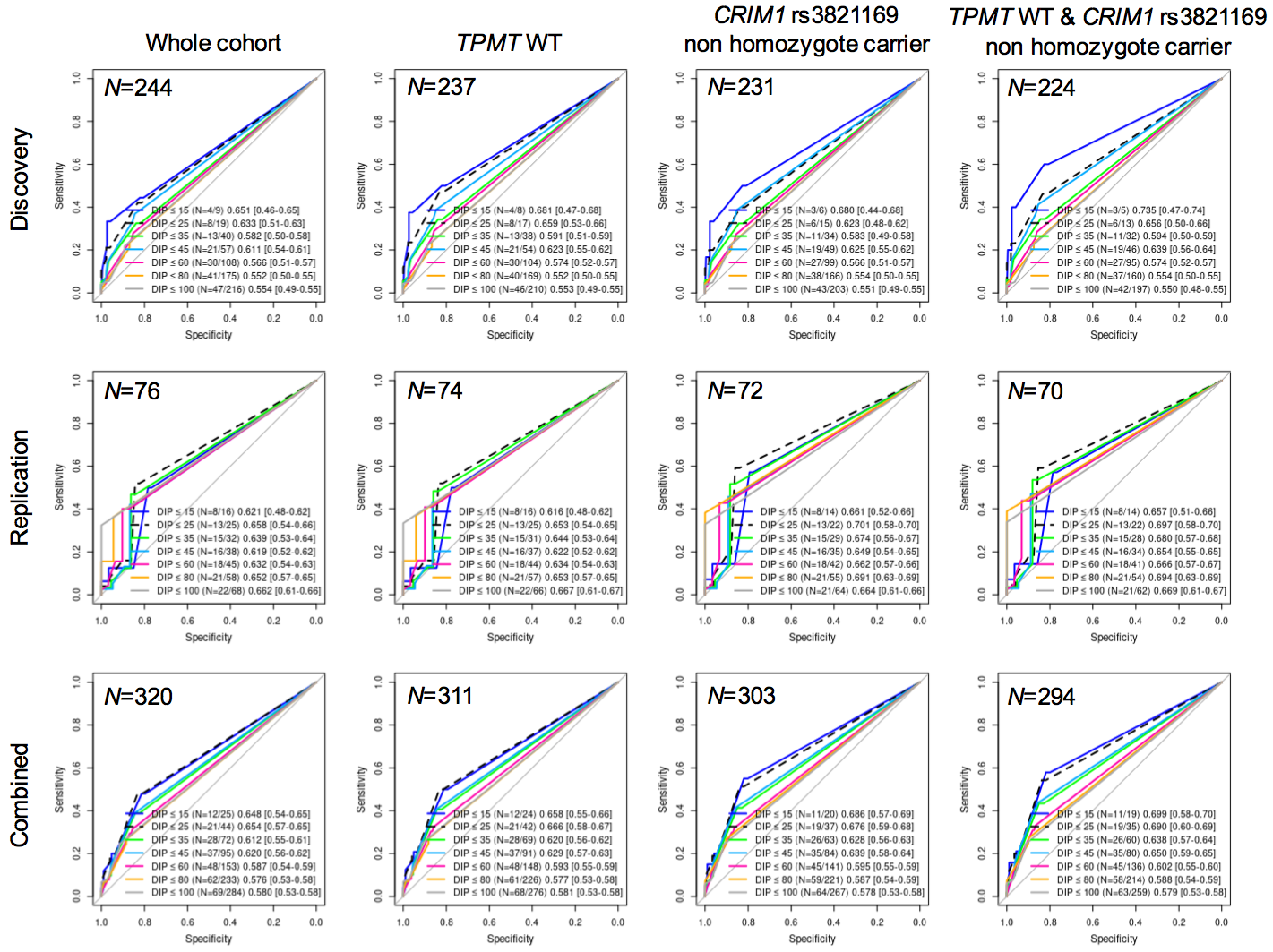


**Figure S6. Prediction accuracy of GVB*^TPMT^* for thiopurine toxicity in pediatric ALL subjects.** Diagnostic accuracies were measured by AUCs with or without controlling for the effect of other confounding variants across different cutoffs of the tolerated last-cycle 6-mercaptopurine dose intensity percentage (%) in discovery, replication, and combined cohorts.


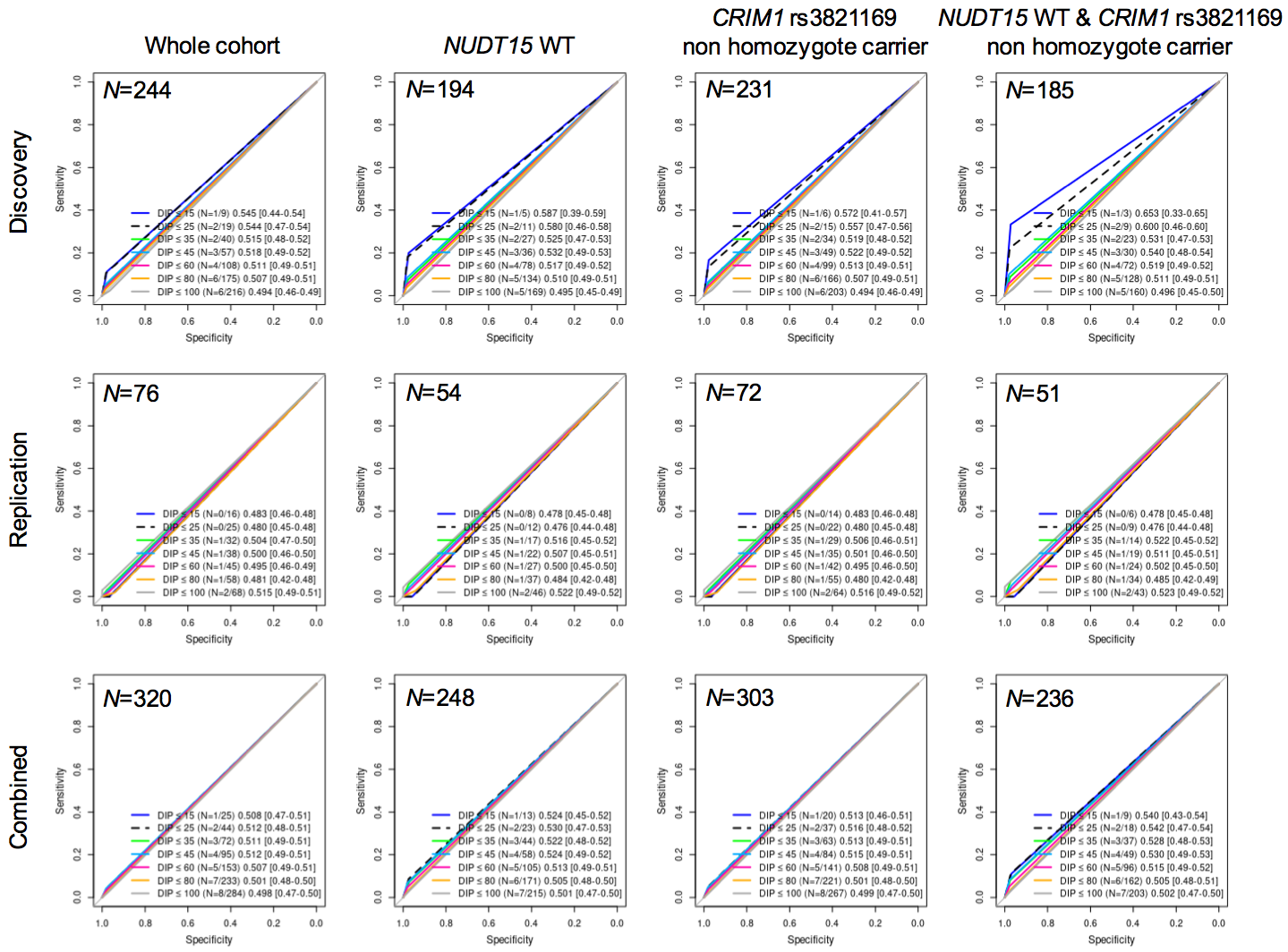


**Figure S7. Youden’s index to find the optimal thresholds for GVB*^NUDT15,TPMT^* and GVB*^NUDT15,TPMT,CRIM1^*.**

**
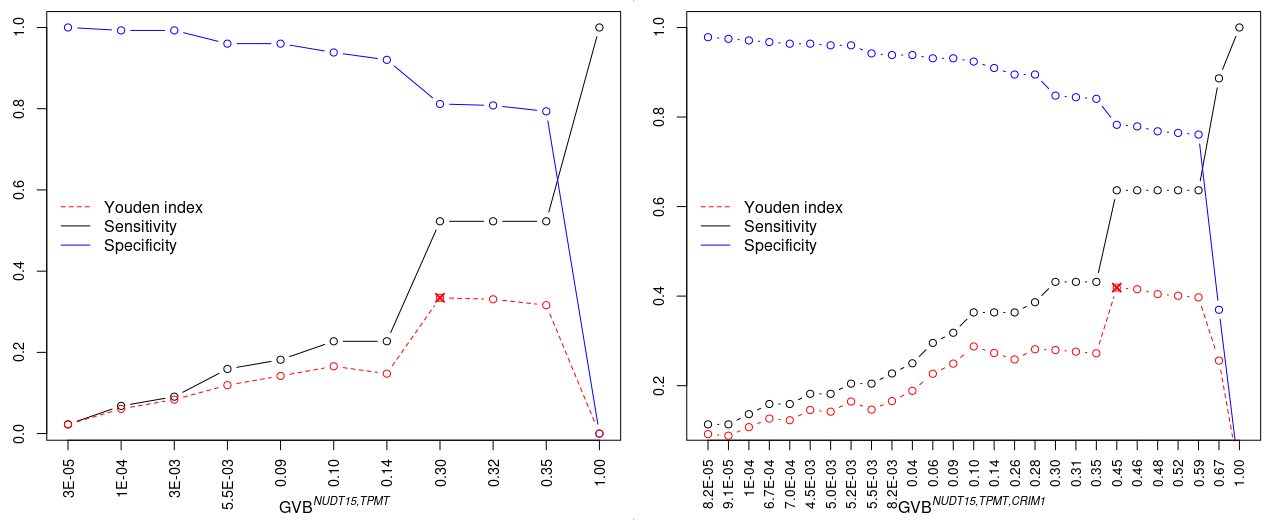
**

**Figure S8. Comparison of *CRIM1* mRNA expression levels of rs3821169 carriers and noncarriers in hematopoietic and lymphoid tissue.**


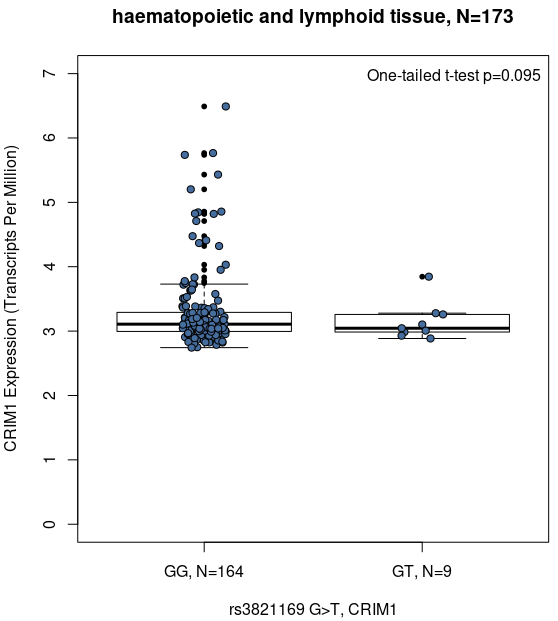


**Figure S9. Results of Sanger sequencing for the two *NUDT15* variants identified via whole exome sequencing.**

**
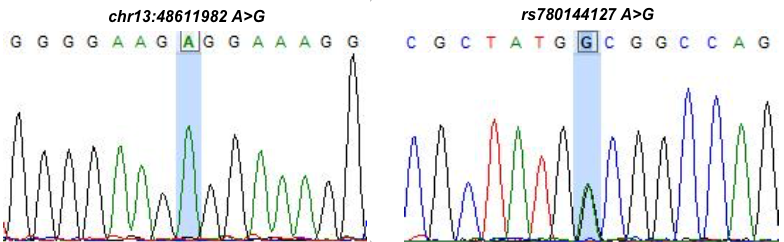
**
